# Synergistic combination of baicalein and rifampicin against *Staphylococcus aureus* biofilms

**DOI:** 10.3389/fmicb.2024.1458267

**Published:** 2024-08-06

**Authors:** Rajeshwari Muniyasamy, I. Manjubala

**Affiliations:** School of Biosciences and Technology, Vellore Institute of Technology, Vellore, India

**Keywords:** *Staphylococcus aureus*, antibiotic resistance, baicalein, rifampicin, synergistic effect

## Abstract

*Staphylococcus aureus*, a Gram-positive bacterium, is a predominant pathogen associated with various infections. The rapid emergence of antibiotic resistance has intensified the challenge of managing fracture-related infections in severe osteoporotic patients. Rifampicin, a potent antimicrobial agent employed against fracture and implant-related infections, necessitates combination therapies due to its susceptibility to antibiotic resistance. In this study, we explored the potential of baicalein, a bioactive flavonoid from *Oroxylum indicum* and *Scutellaria baicalensis*, in combination with rifampicin against *S. aureus* biofilms *invitro*. The minimum inhibitory concentration of baicalein and rifampicin were determined as 500 μg/mL and 12.5 ng/mL respectively. The synergistic activity of baicalein and rifampicin was determined by the fractional inhibitory concentration index (FICI) using checkerboard assay. The results showed the FICI of baicalein and rifampicin was lesser than 0.5, demonstrating synergistic effect. Furthermore, the efficacy of baicalein and rifampicin, both individually and in combination, was evaluated for biofilm inhibition and eradication. Scanning electron microscopy and confocal laser microscopy also confirmed that the synergistic combinations effectively removed most of the biofilms and partially killed pre-formed biofilms. In conclusion, the findings demonstrate that baicalein is as effective as rifampicin in inhibiting and eradicating *S. aureus* biofilms. Their combination exhibits synergistic effect, enhancing their bactericidal effect in completely eradicating *S. aureus* biofilms. The findings of this research underscore the research potential of combining baicalein and rifampicin as a novel therapeutic strategy against *S. aureus* biofilms, offering a promising direction for future research in the treatment of fracture-related *S. aureus* infections.

## 1 Introduction

*Staphylococcus aureus*, a Gram-positive bacterium is an opportunistic pathogen implicated in a spectrum of human infections, from minor skin infections to severe toxin-associated invasive diseases (Idrees et al., 2021). It has been recognized as one of the most common pathogen for nosocomial infections resulting in increased risks of morbidity and mortality in patients (Kong et al., 2018). *S. aureus* is known for developing rapid resistance to antibiotics, primarily driven by antibiotic selection pressure and horizontal gene transfer (Mun et al., 2013). *S. aureus* resistance is also largely exerted due to its ability to adhere and produce biofilms. Biofilms are complex, multilayered extracellular polymeric matrices that shelters bacterial communities. These biofilms shield the bacteria from most antibiotics, leading to an increase in strain resistance by up to 1,500-fold (Wu et al., 2024). The acquisition of resistance to multiple antibiotics renders infections caused by *S. aureus* particularly challenging to manage and treat (Tuon et al., 2023). One such life-threatening condition implicated by *S. aureus* biofilms is fracture-related infections (FRI). Recent findings indicate a rising incidence of open fractures in patients over 65 years of age (Court-Brown et al., 2012; Li et al., 2022). In case of severe osteoporotic patients, there has been increased risk of fracture incidences leading to infections in the implant sites, causing implant-related infections. Due to age-related altered immune response, osteoporosis has been identified as an additional risk factor in infection (Zhang et al., 2022). These fracture-related infections contribute to increased healthcare costs, prolonged recovery, surgical complications, and in severe cases, permanent functional impairment or amputation (Gupta et al., 2015; Rupp et al., 2024). The formation of biofilms by *S. aureus* on implants pose a significant challenge, as they can lead to life-threatening conditions if left untreated.

Rifampicin has been clinically used to treat severe *S. aureus* and implant related infections (Thwaites et al., 2018; Zimmerli and Sendi, 2019). However, the use of rifampicin as a monotherapy is generally not recommended due to the potential risks associated with high spontaneous chromosomal mutation frequencies, which could lead to the emergence of resistance in *S. aureus* (Wang et al., 2019). *S. aureus* biofilms present a significant challenge to the monotherapeutic treatment with rifampicin and kill curve analysis also revealed rapid mutation (Ooi et al., 2010). Interestingly, studies have demonstrated that rifampicin-containing combinations are superior to other antibiotic combinations in eradicating *S. aureus* biofilms, both *in vitro* and *in vivo* conditions (Jørgensen et al., 2016).

To address the challenge of treating *S. aureus* biofilms on FRI, extensive research is underway to discover novel compounds or alternate therapeutic methods. Recent studies have demonstrated the effectiveness of plant-based antimicrobial agents in combating pathogenic bacteria without emerging resistance, possibly by exploiting diverse mechanisms of action that can prevent bacterial adaptation (Borges et al., 2016; Mishra et al., 2020). Various natural bioactive compounds, including flavonoids, alkaloids, terpenoids, phenols and essential oils have potential properties in inhibiting biofilm formation and maturation (Samrot et al., 2021; Shrestha et al., 2022). These bioactive compounds, which are secondary plant metabolites, exhibit a range of biological activities and health beneficial effects with remarkable antimicrobial activity and are generally non-toxic. They are shown to possess potent therapeutic responses against resistant microbes when used in combination with antibiotics (Suganya et al., 2022; Bonincontro et al., 2023).

In this context, the integration of rifampicin with phytocompounds, particularly in the treatment of *S. aureus* biofilms, emerges as a promising area of research. Baicalein is one such naturally occurring bioactive flavonoid majorly found in *Oroxylum indicum and Scutellaria baicalensis* (Salleh et al., 2020). It has been used in numerous traditional herbal medicines as a cure against multitude of infections (Hu et al., 2022). Baicalein has been recently proposed for osteoporotic therapy to target sclerostin of canonical Wnt/β-catenin signaling pathway, enhancing osteogenesis (Muniyasamy and Manjubala, 2023, 2024). Baicalein has also been known to enhance the anti-bacterial effect of antibiotics such as tetracycline, gentamicin, ampicillin, linezolid, penicillin and ciprofloxacin against multiple resistant pathogens when used in combination (Fujita et al., 2005; Chan et al., 2011; Liu et al., 2020; Guo et al., 2021). A combination treatment of rifampicin and baicalein has not been reported against *S. aureus* biofilms *in vitro*, underscoring the novelty and potential impact of this study. This work proposes a novel combination of baicalein in conjunction with rifampicin to serve as an effective agent to inhibit the growth of *S. aureus* biofilms.

## 2 Methods

### 2.1 Bacterial strain, culture conditions and compounds

*Staphylococcus aureus* (MTCC737) was purchased from Microbial Type Culture Collection (MTCC) and Gene bank, Institute of Microbial Technology (IMTECH), India. The culture was grown on Trypticase Soy Agar/Broth (TSA/TSB) at 37°C and maintained at 4°C for future use. Baicalein (CAS 491-67-8, >98% purity) was obtained from Tokyo Chemical Industry (TCI), Japan and dissolved in dimethyl sulfoxide (DMSO) to get the stock solution. The final concentration of DMSO used in the experiments was maintained below 0.01%. All the control wells were treated with same amount of DMSO as the treatment groups.

### 2.2 Determination of minimum inhibitory concentration

The MIC of baicalein and rifampicin was determined using the broth microdilution method according to Clinical Laboratory Standards Institute guidelines (CLSI, 2023). Briefly, 100 μL of cation adjusted Mueller Hinton Broth (MHB) was added to the wells of a 96-well microtiter plate. One hundred microliters of baicalein/rifampicin was aliquoted to the first column of the well and it was serially diluted. The concentrations of baicalein and rifampicin were between 2,000 to 3.9 μg/mL and 100 to 0.18 ng/mL. Then 5 μL of culture (OD_600_ = 0.1) was inoculated in the wells and incubated at 37°C without shaking for 18 h.

The MIC values were also confirmed using resazurin assay. Resazurin is a blue dye that is irreversibly reduced by oxidoreductase present in live bacteria to a pink fluorescent substance called resorufin, which acts as an indicator of bacterial growth. Following incubation of 18 h, 20 μL of 0.01% resazurin solution were added to the wells and further incubated in dark for 3 h (Elshikh et al., 2016). A change in color from blue to pink indicated bacterial growth. The experiments were performed in triplicates and repeated three times independently.

### 2.3 Biofilm inhibition assay

The biofilm inhibition assay was performed in 96-well microtiter plate and quantified using crystal violet assay as described (Stepanović et al., 2007; Song Z. M. et al., 2021). One hundred microliters of bacterial inoculum (OD_600_ = 0.1) mixed with different concentrations of baicalein and rifampicin (1/2 × MIC, MIC, 2 × MIC and 4 × MIC) was aliquoted into the wells and incubated at 37°C for 24 h. After incubation, the planktonic cells were removed by washing the wells thrice gently with 1X PBS. The washed biofilms were stained with 0.5% crystal violet (CV) stain for 15 min at room temperature. The unbound CV was removed by washing with 1X PBS and air-dried. The staining was then dissolved in 100% ethanol and the absorbance was measured at 570 nm. The experiments were performed in triplicates and repeated three times independently.

### 2.4 Biofilm eradication assay

The biofilm eradication assay of baicalein and rifampicin on the 24 h pre-formed biofilm was performed in 96-well microtiter plate as described (Nair et al., 2016). Ten microliters of inoculum (OD_600_ = 0.1) was added to 90 μL of TSB and incubated at 37°C overnight for 24 h. The planktonic cells were then removed by rinsing with 1X PBS. One hundred microliters of different concentrations of baicalein and rifampicin (1/2 × MIC, MIC, 2 × MIC, 4 × MIC, 8 × MIC and 16 × MIC) was added to the wells and incubated at 37°C for another 24 h. After the incubation, the planktonic cells were removed with 1X PBS and the biofilms were stained with CV as described above. The experiments were performed in triplicates and repeated three times independently.

### 2.5 Checkerboard assay

Synergistic efficacy of baicalein and rifampicin was performed by checkerboard microdilution assay. Two-fold serial dilution of baicalein (2,000 to 15.6 μg/mL) was combined with rifampicin (200 to 0.18 ng/mL) to a volume of 100 μL with an inoculum of (OD_600_ = 0.1) in MHB. The microplates were incubated at 37°C for 18–20 h. After incubation, 20 μL of resazurin solution was added to the wells and incubated for 3 h. Baicalein or rifampicin alone were taken as positive controls and untreated cells were taken as negative control. Fractional inhibitory concentration index (FICI) was calculated by observing the highest dilution concentration of antibiotic combination that permits no visible growth. The experiments were performed in triplicates and repeated three times independently.


$$FIC\;\mathrm{of}\;\mathrm{baicalein}\;\mathrm{and}\;\mathrm{rifampicin}=\frac{\mathrm{MIC}\;\mathrm{of}\;\mathrm{baicalein}}{\;\mathrm{MIC}\;\mathrm{of}\;\mathrm{baicalein}\;\mathrm{and}\;\mathrm{rifampicin}\;\mathrm{combined}}+\frac{\mathrm{MIC}\;\mathrm{of}\;\mathrm{rifampicin}}{\mathrm{MIC}\;\mathrm{of}\;\mathrm{baicalein}\;\mathrm{and}\;\mathrm{rifampicin}\;\mathrm{combined}}$$

FICI was interpreted as ≤0.5—synergism; 0.5 < FICI ≤ 4—no effect; FICI >4—antagonism.

### 2.6 Time-kill growth curve assay

Time-kill assays were performed in triplicates as described in the CLSI guidelines (2015). Briefly, different concentrations of baicalein (MIC, 4 × MIC), rifampicin (MIC, 4 × MIC), synergistic combinations of baicalein and rifampicin (FIC, 4 × FIC) were prepared with an inoculum of (OD_600_ = 0.1) in 12-well plates and incubated at 37°C. Viable colony count were determined for predetermined time points (0, 4, 8, 12 and 24 h) using spread plate method. Time-kill curves were constructed using the viable count of CFU/mL against time.

### 2.7 Activity of synergistic combinations against *Staphylococcus aureus* biofilm

The synergistic combinations were tested for their inhibitory effect towards biofilm formation and pre-formed biofilm eradication of *S. aureus*. As described above, 100 μL of bacterial inoculum was mixed with synergistic combinations of baicalein and rifampicin (1/2 × FIC, FIC, 2 × FIC, 4 × FIC and 8 × FIC) in 96 well microtiter plate and incubated at 37°C for 24 h. After the incubation, the wells were washed, stained with CV and absorbance was measured at 570 nm. For pre-formed biofilm eradication, 100 μL of inoculum (OD_600_ = 0.1) was added to 96-well plate and incubated overnight. After the incubation, the planktonic cells were washed twice with 1X PBS. One hundred microliters of baicalein and rifampicin synergistic combinations (1/2 × FIC, FIC, 2 × FIC, 4 × FIC and 8 × FIC) were added to the pre-formed biofilms and further incubated for 24 h. The wells were then washed, stained and absorbance was measured at 570 nm.

### 2.8 Scanning electron microscopic assay of *Staphylococcus aureus* biofilms

The bacterial suspension (OD_600_ = 0.1) containing different synergistic concentrations of baicalein and rifampicin (FIC, 2 × FIC and 4 × FIC) were added to a 6-well plate with coverslips. After incubation at 37°C for 24 h, the coverslips were washed thrice with 1X PBS to remove the planktonic bacteria. Then the coverslips were placed in methacarn solution (60% v/v methanol, 30% chloroform and 10% glacial acetic acid) overnight, followed by dehydration with gradient concentrations of ethanol solution (10–100%, 20 min each). The wells were air dried and the coverslip samples were sputter coated and evaluated by SEM (ZEISS EVO18, Germany).

### 2.9 Confocal laser scanning microscopy of pre-formed *Staphylococcus aureus* biofilms

*S. aureus* biofilms were grown overnight in a 6-well plate. To the pre-formed biofilms, synergistic concentrations of baicalein and rifampicin (FIC, 2 × FIC, 4 × FIC and 8 × FIC) were added and incubated overnight. Following the incubation, planktonic bacterial cells were removed and the biofilms were washed twice with sterile saline. Acridine orange (AO) and propidium iodide (PI) dyes were added to the wells and incubated in dark for 20 min. The excess dye was washed and the biofilms were observed using a 40× magnification on CLSM (Fluoview Fv3000, Olympus, Japan).

### 2.10 Statistical analysis

All the experiments were performed in triplicates and the data were represented as the mean ± standard deviation (SD). One-way analysis of variance (ANOVA) followed by Bonferroni correction was used to determine the statistical significance of the data obtained in this study. Differences were considered statistically significant if *p* < 0.05. All the statistical analysis was carried out in GraphPad Prism 8.0 software.

## 3 Results

### 3.1 Minimum inhibitory concentration of baicalein and rifampicin against *Staphylococcus aureus*

**Figure 1:**
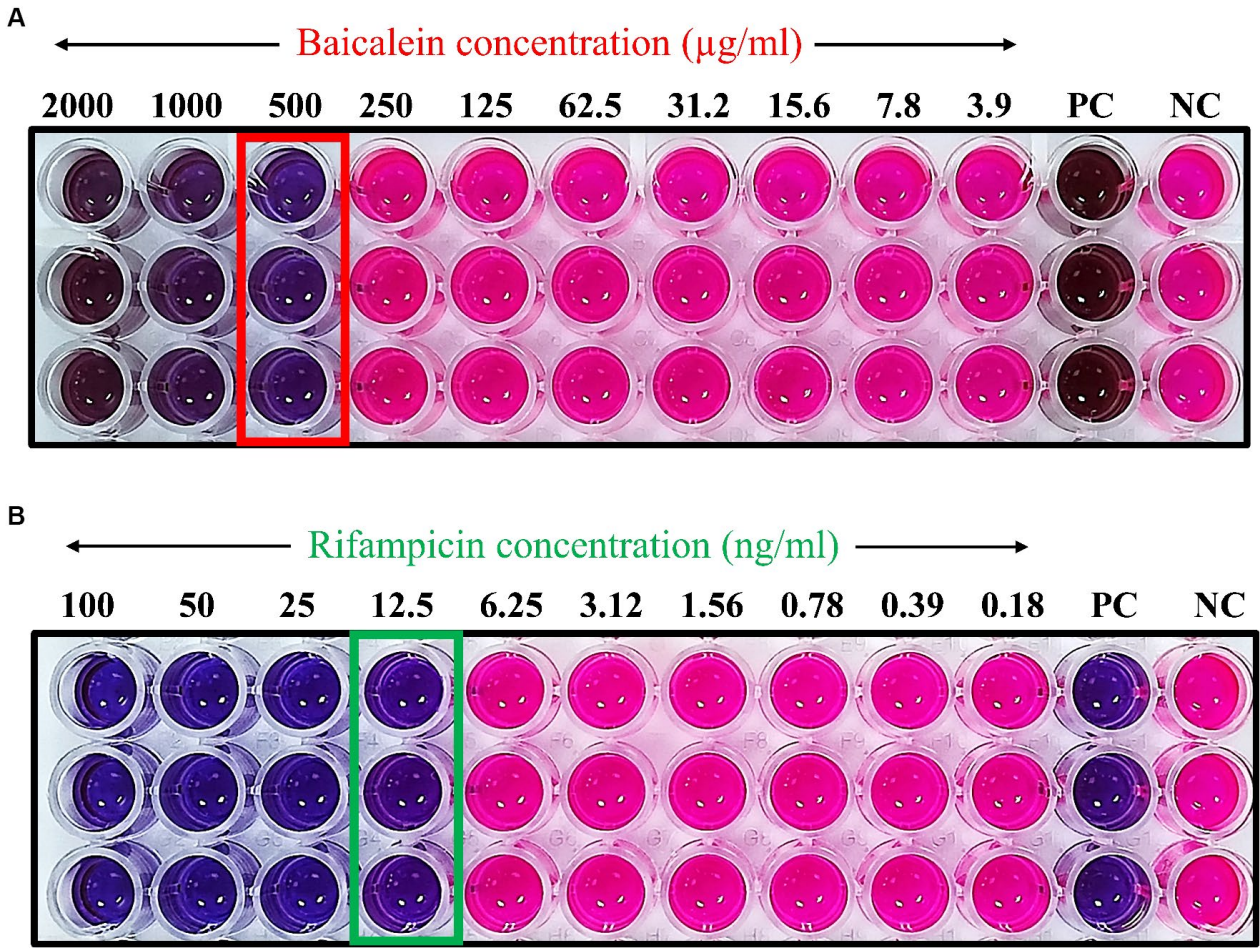
Determination of minimum inhibitory concentration of **(A)** baicalein and **(B)** rifampicin using resazurin assay. The resazurin solution, employed as a bacterial growth indicator, undergoes a color transition from blue to pink in the presence of viable bacteria. The absence of color change at concentrations of 500 μg/mL for baicalein and 12.5 ng/mL for rifampicin signifies the respective MIC values. Concentrations of 3,000 μg/mL for baicalein and 200 ng/mL for rifampicin were utilized as positive controls (PC).

The minimum inhibitory concentration (MIC) was determined using a microdilution assay to identify the least concentration of the drug required to inhibit the visible growth of *S. aureus*. The MIC for baicalein was found to be 500 μg/mL, while for rifampicin it was 12.5 ng/mL. These MIC values were further validated by resazurin assay. This assay serves as an indicator of bacterial growth, with color transition from blue to pink signifying the presence of bacterial proliferation. As shown in Figure 1, concentrations of baicalein above 500 μg/mL and rifampicin above 12.5 ng/mL resulted in no color change, thereby confirming the MIC values.

### 3.2 *Staphylococcus aureus* biofilm formation inhibition by baicalein and rifampicin

**Figure 2:**
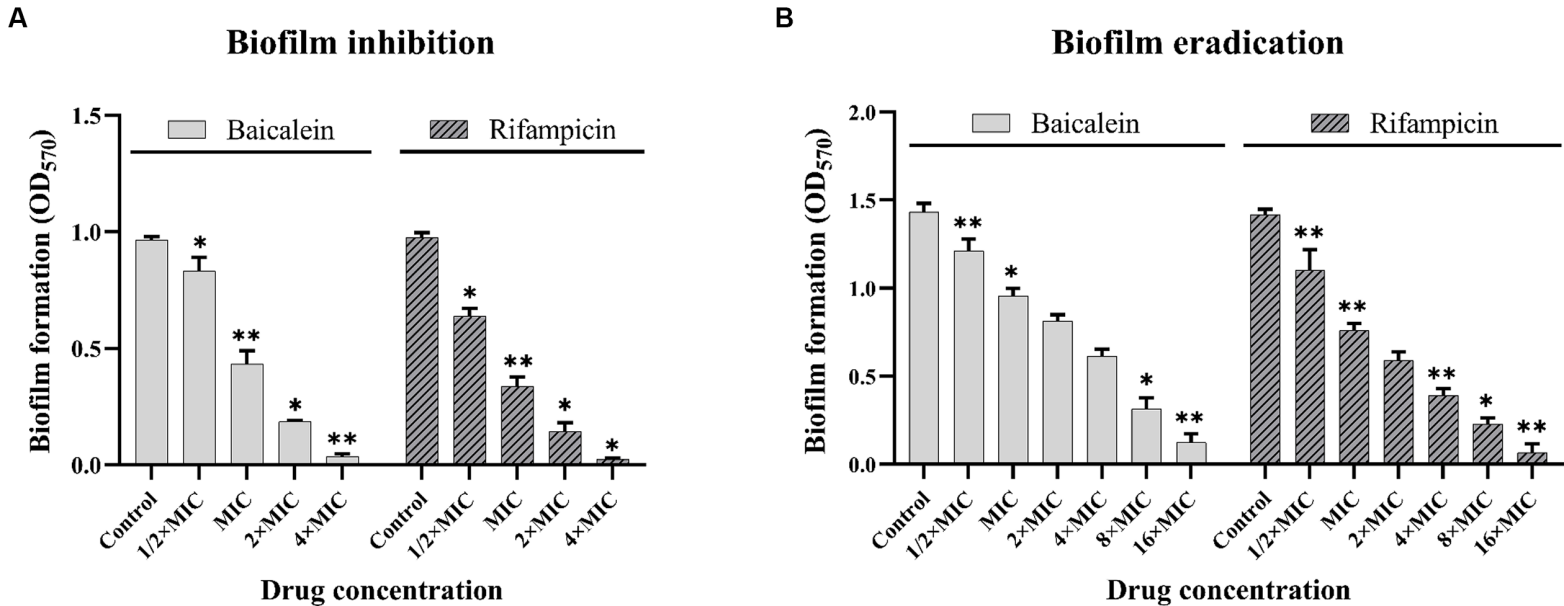
Effect of baicalein and rifampicin on biofilm inhibition and eradication in *S. aureus*. **(A)** Represents the quantitative CV assay results of biofilm inhibition treated with baicalein and rifampicin for 24 h. A complete inhibition of *S. aureus* biofilm is observed at 4 × MIC for both baicalein and rifampicin. **(B)** Represents the quantitative CV assay results of biofilm eradication of 24 h pre-formed biofilms treated with baicalein and rifampicin for 24 h >90% of pre-formed biofilms were eradicated at 16 × MIC for both baicalein and rifampicin. (All values are presented as the mean ± SD and *n* = 9 in each group. Statistical significance is indicated as follows: ^*^p < 0.05 and ^**^p < 0.01, compared to the control group).

To evaluate the effects of baicalein on biofilm formation, *S. aureus* cells were incubated with different concentrations of baicalein and rifampicin in 96-well plates for 24 h. The biofilms were stained with CV and OD was taken at 570 nm. As shown in Figure 2A, baicalein significantly reduced the formation of biofilms *in vitro*. A dose-dependent decrease in biofilm was observed at lower concentrations, culminating in complete inhibition at higher concentrations. 50% reduction of biofilm can be seen at MIC concentration and complete inhibition can be seen at 4 × MIC concentration. Baicalein also performed similar to the anti-staphylococcus antibiotic rifampicin, which showed >50% of biofilm inhibition at MIC concentration and complete inhibition at 4 × MIC concentration.

### 3.3 Biofilm eradication activity of baicalein and rifampicin

The efficacy of baicalein to remove pre-formed biofilms of *S. aureus* was evaluated using crystal violet assay. As shown in Figure 2B, baicalein demonstrated strong antibiofilm properties by significantly disrupting the pre-formed *S. aureus* biofilms. Baicalein and rifampicin at 4 × MIC decreased the biomass of pre-formed biofilms by more than 50% and 16 × MIC eradicated the biofilm by >90%. Interestingly, baicalein performed similar to rifampicin in significantly eradicating the biofilm biomass, signifying its eradicating effect against *S. aureus* biofilms.

### 3.4 Checkerboard assay

**Figure 3:**
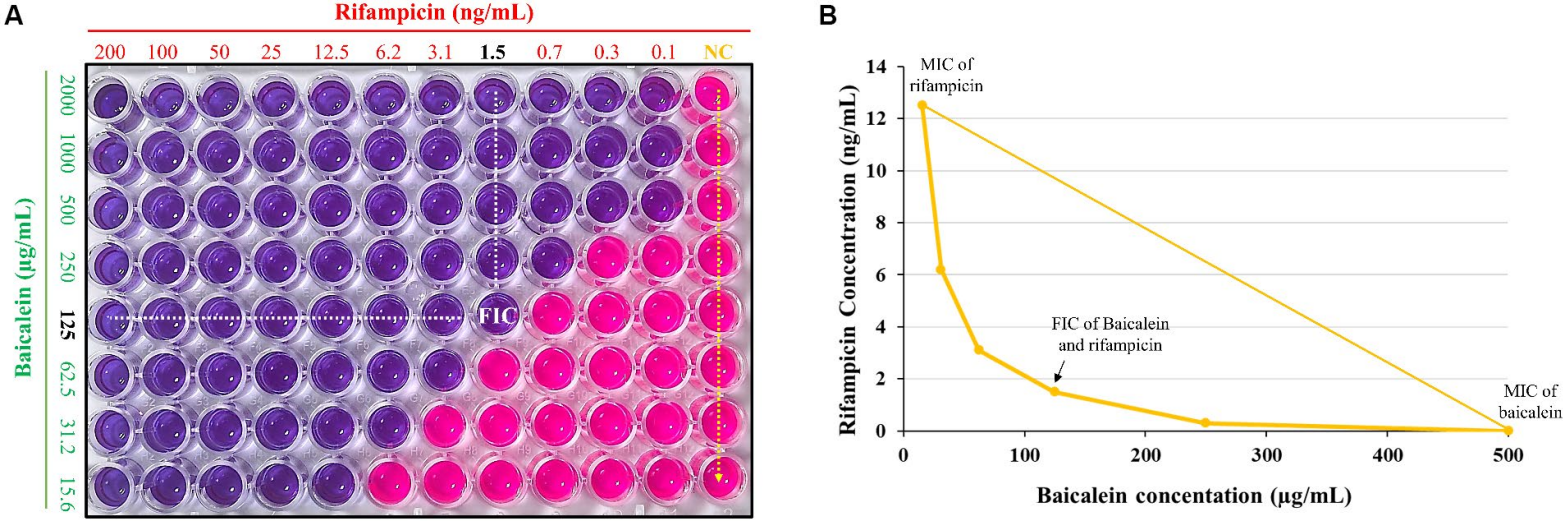
**(A)** Synergistic effect of baicalein and rifampicin against *S. aureus* as determined by checkerboard-resazurin assay. The assay was conducted over a range of concentrations, from 2,000 μg/mL to 1.56 μg/mL for baicalein and from 200 ng/mL to 0.1 ng/mL for rifampicin. **(B)** An isobologram generated from checkerboard assay. The synergistic effect against *S. aureus* was observed at a concentration of 125 μg/mL for baicalein and 1.5 ng/mL for rifampicin.

The synergistic inhibitory effect of baicalein and rifampicin against *S. aureus* was assessed using checkerboard assay for evaluating the interaction between two antimicrobial agents. This was further corroborated using resazurin, for detecting bacterial growth. As shown in Figure 3A, the highest concentrations of baicalein and rifampicin that resulted in no color change, indicating no bacterial growth, were 125 μg/mL and 1.56 ng/mL, respectively. These concentrations represent the minimum inhibitory concentrations (MICs) of the two compounds when used in combination against *S. aureus*. An isobologram was also constructed by plotting the synergistic concentrations from the checkerboard assay (Figure 3B). The Fractional Inhibitory Concentration Index (FICI) was calculated to be 0.37. Given that a FICI value of less than 0.5 is generally considered indicative of a synergistic interaction, our results confirm the synergism between baicalein and rifampicin against *S. aureus*.

### 3.5 Time-kill curve assay

**Figure 4:**
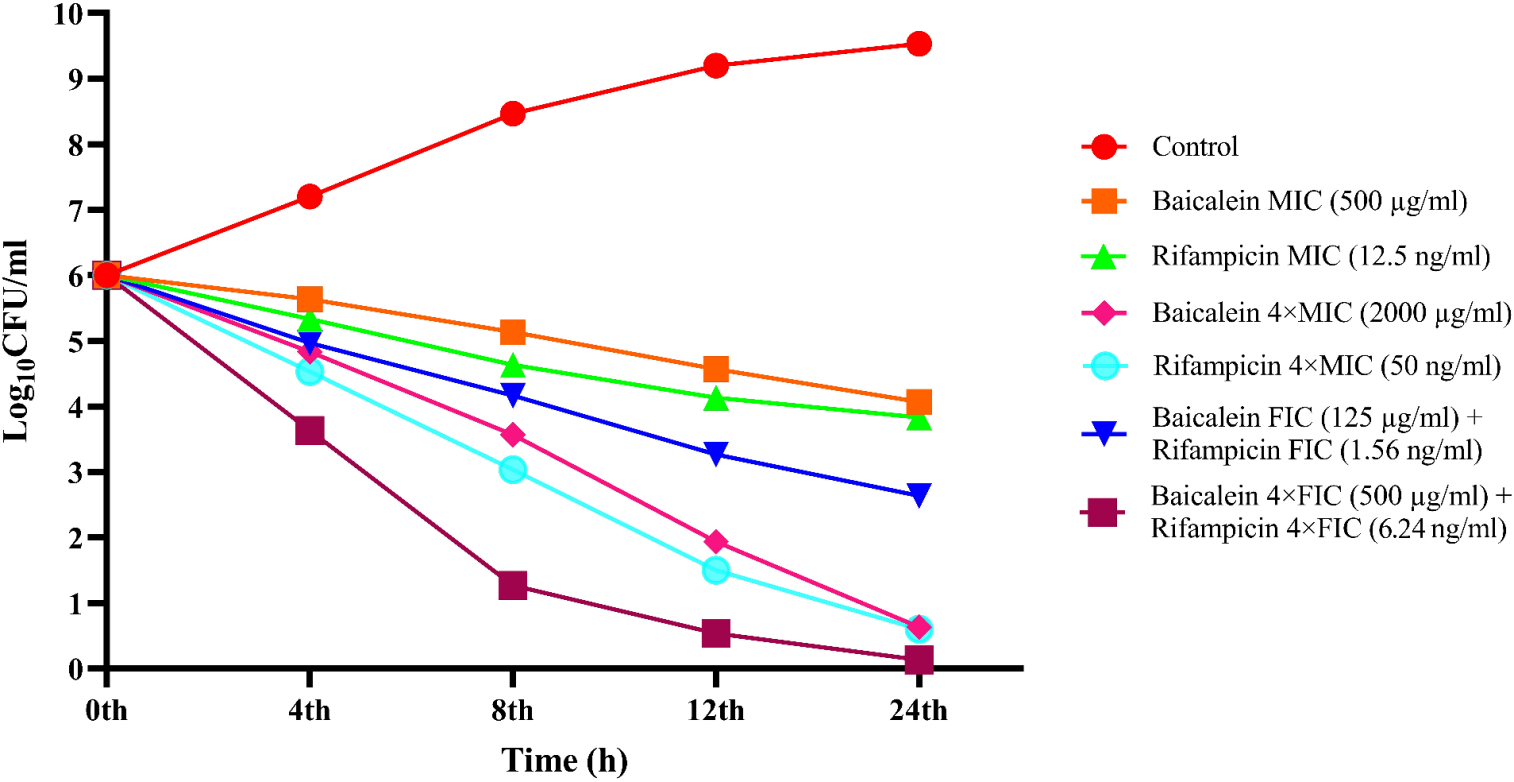
Time-kill curve of *S. aureus* treated with different concentrations of baicalein and rifampicin. Complete inhibition of *S. aureus* was observed at 4 × MIC of both baicalein and rifampicin at 24 h, whereas 4 × FIC resulted in complete growth inhibition at the end of 12 h.

To determine and compare the bactericidal effect of baicalein and rifampicin along with their synergistic combinations, time-kill curve assay was performed to measure the reduction of colony-forming units (CFU) of *S. aureus* against time. As shown in Figure 4, treatment with MIC and FIC of baicalein and rifampicin merely inhibited the proliferation of *S. aureus*, without inducing cell death. However, 4 × MIC of both baicalein and rifampicin demonstrated bactericidal effect, leading to the complete reduction and subsequent death of *S. aureus* after 24 h. The FIC of baicalein and rifampicin caused an over 2 log_10_-fold reduction in bacterial count after 24 h, whereas 4 × FIC of baicalein and rifampicin completely eradicated *S. aureus* population after just 12 h. It can be seen that combination of baicalein and rifampicin can dramatically enhance the bactericidal effect resulting in the lowering of CFUs.

### 3.6 Synergistic combinations of baicalein and rifampicin on *Staphylococcus aureus* biofilm

**Figure 5:**
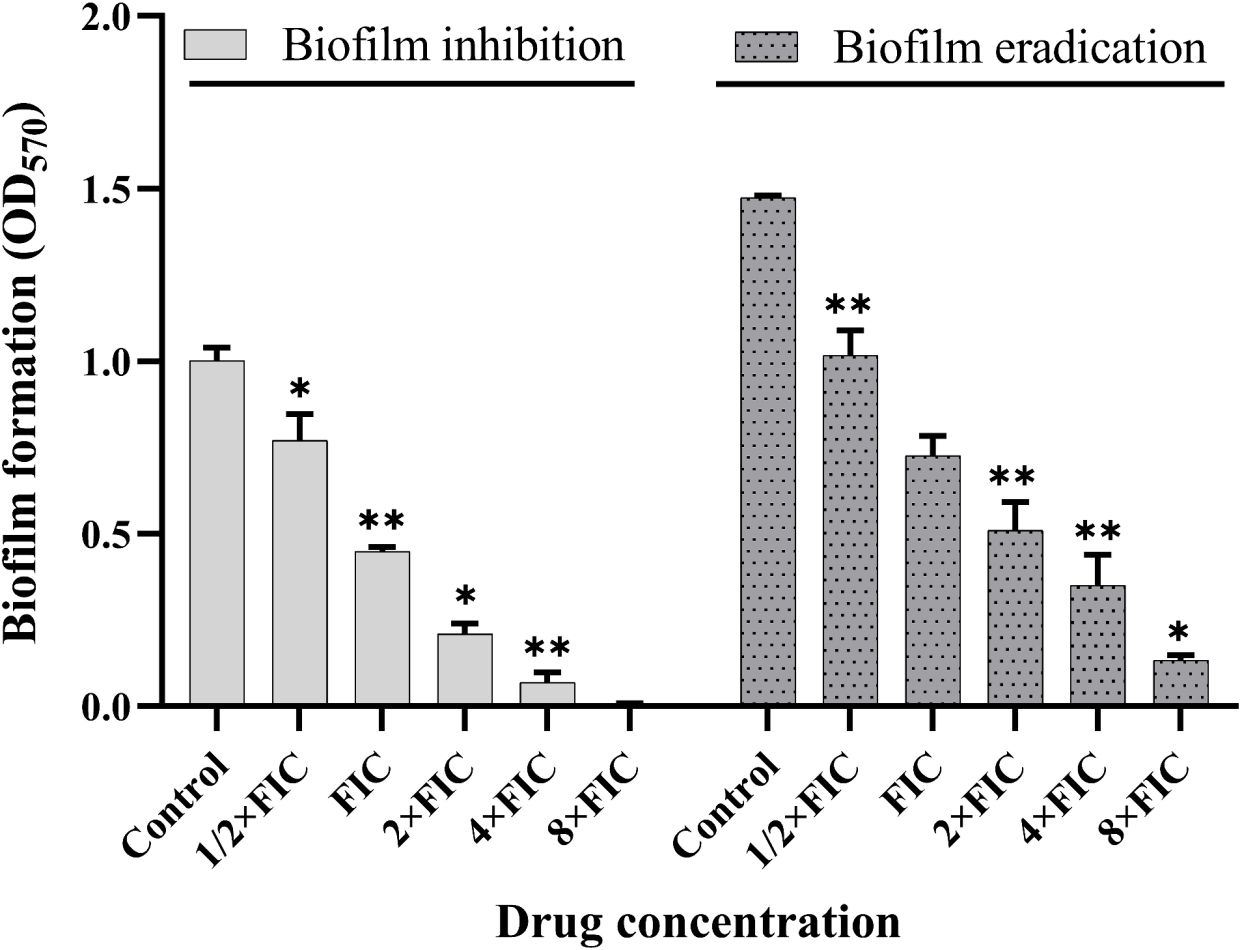
Evaluation of biofilm inhibition and eradication activity of synergistic concentrations of baicalein and rifampicin against *S. aureus*. Biofilm formation was inhibited by more than 90% at 4 × FIC, and a similar eradication rate was achieved for pre-formed biofilms at 8 × FIC. (All values are presented as the mean ± SD and *n* = 9 in each group. Statistical significance is indicated as follows: ^*^p < 0.05 and ^**^p < 0.01, compared to the control group).

To further analyze the synergistic efficacy of baicalein and rifampicin against *S. aureus* biofilms, biofilm inhibition and eradication assays were performed. As shown in Figure 5, the synergistic combinations effectively reduced the biofilm formation of *S. aureus*. 50% of biofilm formation was inhibited with FIC synergistic concentrations (125 μg/mL and 1.56 ng/mL) and maximum inhibition is achieved with 4 × FIC concentration (500 μg/mL and 6.24 ng/mL) of baicalein and rifampicin respectively. Synergistic combinations of baicalein and rifampicin also had appreciable effect on eradicating the pre-formed biofilms effectively. 4 × FIC concentration eradicated the pre-formed biofilms by 50% whereas 8 × FIC concentration eradicated >90% of the pre-formed biofilm.

### 3.7 Microscopic evaluation of *Staphylococcus aureus* biofilms treated with synergistic combinations

**Figure 6:**
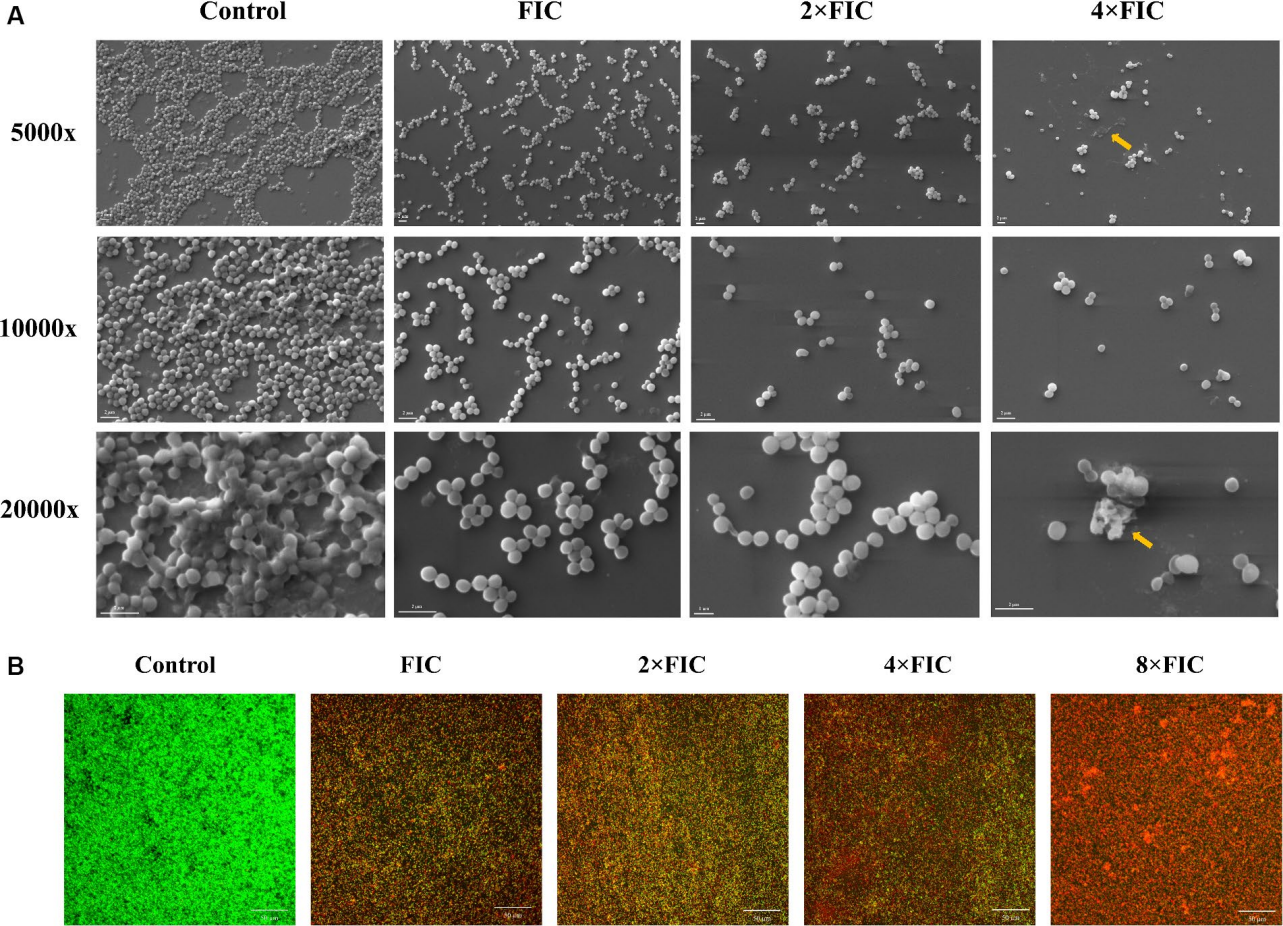
**(A)** Scanning electron microscopy images depicting the inhibitory effect on biofilm formation following treatment with FIC, 2 × FIC, and 4 × FIC of baicalein and rifampicin, compared to the control. Images are presented at magnifications of 5,000×, 10,000×, and 20,000×. Yellow arrows represents damaged cells in the biofilm. **(B)** Confocal laser scanning microscopy images of 24 h pre-formed biofilms treated with FIC, 2 × FIC, 4 × FIC, and 8 × FIC. Biofilms were stained with acridine orange (AO; green) and propidium iodide (PI; red) and imaged at 40× magnification. Scale bars represent 50 μM.

To further characterize the effect of synergistic concentrations of baicalein and rifampicin on *S. aureus* biofilm formation, *S. aureus* was supplemented with various concentrations (FIC, 2 × FIC and 4 × FIC) and allowed to form biofilms on coverslips in a 6-well plate. The morphological characteristics of the biofilm architecture, both treated and untreated, were visualized using SEM following a 24 h incubation period. As shown in the Figure 6A, the untreated control exhibited a substantial multi-layered biofilm structure, characterized by densely packed bacterial colonies. Upon treatment with the synergistic concentrations, the biofilm formation was notably disrupted, with negligible biofilms detected at higher concentrations. The bacterial colonies appeared as a monolayer and were dispersed across the surface. Upon examination at a higher magnification (20,000×), untreated *S. aureus* appeared as large clusters of cells in overlapping layers. In contrast, the synergistic treatment groups appear more scattered, with minimal clumping observed as the concentration increased. At 2 × FIC, the cells appeared sparser and more disrupted, indicating a potential bactericidal effect of the treatment. And 4 × FIC resulted in complete inhibition of biofilm formation in which resulted in no visible cells. These SEM images also corroborate the data from the CV biofilm inhibition assay.

CLSM assay was performed to distinguish between live and dead cells of pre-formed *S. aureus* biofilms after treating with synergistic combinations of baicalein and rifampicin. Dual fluorescent staining was performed with AO/PI, where AO penetrates the cell membrane of live and dead cells and stains green, whereas PI can only penetrate membrane disrupted dead cells, binds to nucleic acids and stains red. As shown in the Figure 6B, the synergistic combinations of baicalein and rifampicin, potentially destroyed the membrane of *S. aureus* biofilm as the dead cells in the biofilms increased in a dose-dependent manner. CLSM results indicates that synergistic combinations could eradicate the pre-formed biofilms via membrane disruption.

## 4 Discussion

In the rapidly evolving field of antimicrobial research, the exploration of novel therapeutics is of paramount importance, particularly in the context of increasing antibiotic resistance. The emergence of antibiotic resistance in *S. aureus* fracture-related infections threatens osteoporotic patients worldwide and the efficacy of antimicrobials used in the treatments are decreasing, exhibiting little or no effect if used alone (Cheung et al., 2021; Mlynarczyk-Bonikowska et al., 2022). Rifampicin, a renowned antibiotic for treating implant-related infections, is prone to develop resistance *in S. aureus*. This escalating drug resistance highlights the importance for the hunt of novel antimicrobials. Recently studies on plant-derived alternatives revealed the antimicrobial and antibiofilm properties of several novel phytocompounds with multiple mechanism of action that can combat the drug resistance if used in combination with antibiotics (Yong et al., 2019). Baicalein, an important flavonoid of *O. indicum* and *S. baicalensis*, has been used in traditional medicines for treating multiple diseases (Song J. et al., 2021; Gupta et al., 2022; Muniyasamy and Manjubala, 2024). Recently, baicalein has been proposed as a potent anti-osteoporotic agent and studies have also proven the synergistic effect of baicalein with other antibiotics to treat drug resistant pathogens (Liu et al., 2020; Vijayakumar et al., 2021; Güran et al., 2023). In light of these findings, baicalein poses as a suitable bioactive flavonoid that can treat fracture-related infections in combination with rifampicin. Hence, this study focused on identifying the potentiality of baicalein in adjunct with rifampicin against *S. aureus* biofilms.

In this study, we assessed the sensitivity of *S. aureus* against baicalein and rifampicin by calculating the MIC, determined using broth microdilution assay. MIC of baicalein and rifampicin was 500 μg/mL and 12.5 ng/mL respectively, which was further validated by resazurin assay. The study also demonstrated the effectiveness of baicalein in inhibiting biofilm formation, a key virulence factor of *S. aureus*. A dose-dependent decrease in biofilm was observed, with complete inhibition at higher concentrations (4 × MIC). Interestingly, baicalein was found to be as effective as the anti-staphylococcal antibiotic rifampicin in inhibiting biofilm. In addition, baicalein also showed strong antibiofilm properties by significantly disrupting pre-formed *S. aureus* biofilms, similar to rifampicin with <90% eradication of biofilms with 16 × MIC. These results are in accordance with previous studies confirming the antimicrobial and antibiofilm properties of baicalein against *S. aureus* (Chen et al., 2016; Mao et al., 2023).

Drug combinations show better efficacy in enhancing their effectiveness while minimizing side effects and cytotoxicity (Ayaz et al., 2019). In our study, the combinations of baicalein and rifampicin was checked for their synergistic effect against *S. aureus.* The checkerboard assay coupled with resazurin revealed that 125 μg/mL of baicalein and 1.56 ng/mL of rifampicin as the FIC values and they were confirmed to have synergistic effect as evidenced by the FIC value <0.5 (i.e., 0.37). Rifampicin owing to its ability to easily penetrate the cells enhanced the effectiveness of baicalein, suggesting that this combination could be a promising strategy for increased bactericidal effect against *S. aureus*.

The time-kill curve assay further assessed the time-dependent bactericidal effect of baicalein and rifampicin, both individually and in combination. Treatment with 4 × MIC of both agents led to the complete reduction and subsequent death of *S. aureus* after 24 h, whereas 4 × FIC of baicalein and rifampicin completely eradicated the *S. aureus* population after just 12 h. This enhanced killing efficacy with less time span confirmed the potential of the combinatorial treatment. The synergistic combinations of baicalein and rifampicin were also effective in inhibiting biofilm formation, 4 × FIC inhibited more than 90% of biofilm formation, whereas 90% of pre-formed biofilms were eradicated by 8 × FIC. Various studies have also demonstrated that rifampicin-based combination regimens with phytocompounds exhibits enhanced effectiveness in targeting and eradicating *S. aureus* biofilms than individual treatment (Müller et al., 2013; Liu et al., 2018).

Microscopic visualization of FICs treatment of baicalein and rifampicin induced alterations on biofilm architecture of *S. aureus* was performed through SEM analysis. The biofilm without baicalein was observed to form thick aggregates, while the biofilms exposed to FICs was gradually decreased and disrupted. This result was in accordance with previous studies that highlighted the biofilm destruction using phytocompounds. Gu et al. (2022) and Xu et al. (2023), observed the dose-dependent decrease of *S. aureus* biofilm as the treatment with phytocompounds such as geraniol and punicalagin increased respectively. Morphological observation of pre-formed *S. aureus* biofilms using CLSM revealed that synergistic treatment groups effectively killed the 24 h old biofilms in a dose-dependent manner.

Our findings demonstrate that the synergistic combination of baicalein and rifampicin exhibits superior efficacy in inhibiting the growth of *S. aureus* and its biofilm formation compared to their individual antimicrobial effect. This synergistic interaction between baicalein and rifampicin significantly enhances their antimicrobial potency, thereby outperforming the effects observed when used alone. The enhanced efficacy of the combination therapy can be attributed to the complementary mechanisms of action of baicalein and rifampicin, which collectively target multiple aspects of the bacterial life cycle, including growth and biofilm formation. Since the mechanism of *S. aureus* biofilm formation is complex, the effect of baicalein and its synergistic combination with rifampicin on the expression of genes for biofilm formation needs to be determined in the future.

## 5 Conclusion

In conclusion, this study provides compelling evidence for the antimicrobial and antibiofilm properties of baicalein against *S. aureus*, both individually and in combination with rifampicin. Our findings suggest that synergistic combinations of baicalein and rifampicin has superior antibiofilm properties in inhibiting *S. aureus* biofilms than used alone. These results underscore the potential benefits of employing combinational therapies with phytocompounds in the clinical management of fracture-related *S. aureus* infections, and highlight the need for further research into the optimization of such therapeutic strategies. However, further studies are needed to elucidate the exact mechanisms underlying the observed effects and to evaluate the *in vivo* efficacy and safety of these treatments.

## Data availability statement

The original contributions presented in the study are included in the article, further inquiries can be directed to the corresponding author (i.manjubala@vit.ac.in).

## Author contributions

RM: Conceptualization, Data curation, Formal analysis, Investigation, Methodology, Project administration, Resources, Validation, Visualization, Writing – original draft, Writing – review & editing. IM: Conceptualization, Data curation, Formal analysis, Methodology, Project administration, Resources, Supervision, Validation, Visualization, Writing – review & editing.
